# Comparing a Social and Communication App, Telephone Intervention, and Usual Care for Diabetes Self-Management: 3-Arm Quasiexperimental Evaluation Study

**DOI:** 10.2196/14024

**Published:** 2020-06-02

**Authors:** Ching-Ju Chiu, Yung-Chen Yu, Ye-Fong Du, Yi-Ching Yang, Jou-Yin Chen, Li-Ping Wong, Chanuantong Tanasugarn

**Affiliations:** 1 Institute of Gerontology College of Medicine National Cheng Kung University Tainan Taiwan; 2 Department of Nursing National Cheng Kung University Hospital Tainan Taiwan; 3 Division of Endocrinology and Metabolism Department of Internal Medicine National Cheng Kung University Hospital Tainan Taiwan; 4 Department of Family Medicine National Cheng Kung University Hospital Tainan Taiwan; 5 Department of Public Health College of Medicine National Cheng Kung University Tainan Taiwan; 6 Department of Social and Preventive Medicine Faculty of Medicine University of Malaya Kuala Lumpur Malaysia; 7 Faculty of Public Health Mahidol University Bangkok Thailand

**Keywords:** diabetes, self-management, depression symptoms, distress, middle-aged and older adults

## Abstract

**Background:**

Many technology-assisted innovations have been used to manage disease. However, most of these innovations are not broadly used by older adults due to their cost. Additionally, disease management through technology-assisted innovations has not been compared with other interventions.

**Objective:**

In this study, we tested the employment of a free and widely used social and communication app to help older adults with diabetes manage their distress and glycemic control. We also compared the effectiveness of the app with 2 other methods, namely telephone and conventional health education, and determined which subgroup experiences the most effects within each intervention.

**Methods:**

Adults aged ≥50 years with type 2 diabetes were recruited from Southern Taiwan (N=231) and were allocated to different 3-month interventions. Informed consent was obtained at the Ministry of Science and Technology and approved by the National Cheng Kung University Hospital Institutional Review Board (No. A-ER-102-425).

**Results:**

Participants in the mobile-based group had significant reductions in hemoglobin A1c compared with the telephone-based and usual care groups (mean changes of –0.4%, 0.1%, and 0.03%, respectively; *P*=.02). Diabetes-specific distress decreased to a greater extent in the mobile-based group compared to the other 2 groups (mean changes of –5.16, –3.49, and –2.44, respectively, *P*=.02). Subgroup analyses further revealed that the effects on reducing blood glucose levels in the social and communication app groups were especially evident in patients with lower distress scores, and diabetes-related distress was especially evident in participants who were younger than 60 years or had higher educational levels.

**Conclusions:**

The findings of this study inform more flexible use of social and communication apps with in-person diabetes education and counselling.

## Introduction

Existing interventions for people with diabetes generally involve group or individual counseling for self-management education and support [1-3]. The use of technology to collect data through the internet to manage disease is increasing; many such studies focus on diabetes, including monitoring glucose or vital signs through mobile apps or websites, messaging reminders through email or SMS text messaging for self-care skills, and improving emotional status [4-8]. In recent years, mobile phones have become an increasingly important platform for the delivery of health interventions; they can be used to help individuals manage their health and disease condition [9-13]. Web-based structured integrated care has been employed by several research teams to provide information for diabetes education, record blood glucose readings, and provide personal feedback [14-16]. Many mobile apps have been developed for diabetes care. Better outcomes were observed for mobile apps that provided 2-way communication between people with diabetes and their health care team [17,18]. However, most of these interventions require the development of a structured website or a diabetes-specific mobile app; these are not translated into many languages, which limits their universal use [19].

Currently, 53% of adults age 65 and older use the Internet in Taiwan [20], and one third of older persons who are online use social networking sites such as Facebook, LinkedIn, and LINE, which were listed as age-friendly apps in 2012 [21]. Some traditional technologies (eg, telephones, video game consoles) have been utilized to conduct health-related interventions. New technologies such as exoskeleton human body posturizers [22], the Nintendo Wii console [23], and virtual reality gaming [24] have also been investigated to assist older people in improving their balance and have been shown to decrease both the fear of falling and the number of falls. For diabetes control apps in Taiwan, the National Health Insurance program developed an internet platform called My Health Bank. My Health Bank provides patients’ medical records over the past 3 years; patients can use it to record their own health data, such as BMI and blood test results, including fasting plasma glucose, hemoglobin A1_c_ (HbA_1c_), and lipid profiles. Physicians can quickly understand their patients’ physical condition, treatment status, and medical examinations through this app [25]. Moreover, many studies have explored the use of new technologies such as websites or mobile apps to help with disease or health management, such as smoking cessation [26,27], medication management [28], and prevention of excessive gestational weight gain and associated maternal and child health consequences [29]. However, few existing studies have focused on the effects of existing, freely available communication apps (eg, Facebook Messenger, LINE, WhatsApp Messenger) among middle-aged and older adults; therefore, the effectiveness of social networking interventions to address the health issues of middle-aged and older adults is not certain. One of the most age-friendly and easy-to-use social network applications is known as LINE; it does not restrict the learning pace, time, or space of learners, and users also benefit from the interaction in this mobile app [30]. In addition, information about diabetes self-management can be easily transmitted through text messages, photographs, and short videos to enable people to use this information in daily life. LINE is one of the most popular apps for social communication in Taiwan [31]. However, whether this tool can be employed in self-management education and support to help older adults manage their diabetes is unknown.

According to the American Association of Diabetes Educators (AADE), 7 behavioral indicators [4] are critical to enhancing the self-care abilities of people with diabetes and to helping them reach their optimal goal of glycemic control through education and various support resources. These indicators, called the AADE7 Self-Care Behaviors, are (1) healthy eating, (2) being active, (3) monitoring, (4) taking medication, (5) problem solving, (6) reducing risks, and (7) healthy coping. The results of one study indicated that most diabetes apps do not adhere to more than 2 AADE7 self-care behavior guidelines [32]. Therefore, the aims of this study were to test 3 intervention modes that were developed based on the AADE7 self-care behavior guidelines, including mobile-based, telephone-based, and usual care interventions. We compared the impact of the mobile-based, telephone-based, and usual care interventions on diabetes-related distress, depressive symptoms, and glycemic control in middle-aged and older adults living in the community. We also compared the degrees of acceptance within the 3 groups to determine which subgroup experienced the most effects.

## Methods

This study was a 3-arm quasiexperimental design. We recruited participants aged ≥50 years with type 2 diabetes through a medical chart review. Exclusion criteria were people with renal dysfunction (creatinine ≥1.5 milligrams per deciliter) or who were receiving dialysis treatment. People who were diagnosed with dementia, cognitive impairment, or major depression were also excluded. We recruited our participants from the endocrine and family medicine clinics of a medical center in Southern Taiwan. Informed consent was obtained at the Ministry of Science and Technology, and the study was approved by the National Cheng Kung University Hospital Institutional Review Board (No. A-ER-102-425).

In the mobile-based group, participants were required to own a mobile device and to be capable of using the mobile device (n=49). Participants who did not have a mobile device and access to the internet at home were randomized to either the telephone-based interview group (n=91) or the usual care group (n=91).

Each group was provided with diabetes-related self-care information and provided with emotional support through usual care. In the mobile-based intervention group, in addition to the participants’ usual care, we used a communication app called LINE to send multimedia messages about diabetes self-management for 12 weeks. LINE is a free instant communication service. In Taiwan, over 90% of people install LINE on their mobile devices [33]. LINE users can set up their own accounts using their telephone number or email address and can link with friends by telephone number or LINE ID. When the participants were ready to join our study, we checked their telephone number or LINE ID and linked it to our research account, and a welcome message was sent to the individual accounts of the participants. The research team verified whether the messages we sent to each individual account were marked as “read.” The participants were also able to interact with our research team by LINE. Messages were sent 3 times a week, and the participants received an average of 5 messages per week. Structural diabetes health education modules were developed according to AADE7 guidelines. We included 6 of the behavioral indicators in our messages: healthy eating (eg, how to select appropriate food and observe amounts of sweeteners); being active (eg, suitable ways to exercise and precautions related to exercise); monitoring (eg, the standard glucose values before and after a meal); taking medication (eg, what to do if they forgot to take their medicine); (5) problem solving (eg, how to relax and search for health information); and (6) reducing risk (eg, foot care skills). The topics of the weekly messages are shown in Textbox 1 and sample messages are shown in Multimedia Appendix 1.

In the telephone-based intervention group, the participants received 3-4 phone calls lasting 30-60 minutes each from a diabetes health educator. The first call explored the participant’s diet, sleep habits, exercise level, and blood glucose control as well as their general and diabetes-specific health conditions. The people with diabetes were asked to talk about their feelings and lifestyle changes after they were diagnosed with diabetes. The goal was to understand their daily routines, thoughts, worries, related feelings, and behavior. According to the baseline assessment, in the second and subsequent phone calls, the assistants asked the participants to discuss self-care problems related to diabetes and their feelings about the disease in addition to the AADE7 modules. The usual care group received a routine intervention for people with diabetes in hospital, where the participants received 5-10 minutes of usual care (including a nutrition consultation, exercise guidance, medication, and other self-care skills related to diabetes) once every 3 months by family medicine physicians in family medicine outpatient departments and by certified diabetes educators at diabetic health education clinics.

We collected data on the health conditions of the patients, such as the year they were diagnosed with diabetes, body weight, height, anti-diabetic therapy, and recent HbA_1c_ levels through a medical chart review. We reviewed the medical record data related to complications such as diabetic foot, hypertension, and stroke. We also collected self-reported data about sociodemographic characteristics, health behavior, and psychological well-being. Sociodemographic factors included age, sex, education, ethnicity, and marital status. The educational levels were divided into 6th grade and below, grades 7-12, and grades 13 and above. The options in the ethnicity category were Taiwanese, mainlander, Hakka, and aborigine. The possible answers to the question on marital status were widowed, married and living with spouse, and other (divorced, separated, or unmarried). The health behavior variables included the frequency and time of exercise, dietary habits, tobacco use, alcohol use, and the amount of alcohol consumed. These data were collected at baseline and after the intervention.

Diabetes-specific emotional distress was measured using the Problem Areas in Diabetes (PAID) scale. It contains 20 questions; each question describes a problem that a person with diabetes may have. The PAID scale uses a 5-point item scale: 1=Not a problem, 2=Minor problem, 3=Moderate problem, 4=Somewhat serious problem, and 5=Serious problem. A higher PAID score indicates greater emotional distress related to diabetes. The total scale of PAID is 0-100, and the commonly used cutoff score of ≥40 was used to indicate elevated levels of diabetes-specific emotional distress [34].

Depressive symptoms were measured with the Center for Epidemiological Studies Depression Scale (CES-D), which is a self-reported questionnaire. The CES-D contains 20 questions. It has been found to have good validity and reliability for different races and ages [35]. In the pretest, we used the brief CES-D. The brief CES-D contains 10 questions on a 4-point scale to classify the depression status of older persons in the most recent week, with possible answers of “not at all,” “usually,” and “always.” The short-form CES-D scale ranges from 0-30, with a higher CES-D score indicating a higher level of depression. The cutoff scores for depressive symptoms were ≥16 for the full-length questionnaire and ≥10 for the 10-item version for older adults [36,37].

**The topics of the weekly SMS text messages.**

**Week 1. Healthy eating**

- Regular diet and principles of eating out for people with diabetes
- Appropriate food and desserts for people with diabetes
- Awareness of artificial sweeteners
- Low calorie diet education

**Week 2. Healthy eating**

- Recording daily amounts of milk, starch, vegetables, fruits, beans, and meat
- Carbohydrate counting
- Glycemic indices of common fruits and low fat food groups

**Week 3. Being active**

- The importance and principles of exercise for people with diabetes
- Photographs of a variety of exercise positions for middle-aged and older adults, such as warmups and calisthenics

**Week 4. Being active**

- Photographs of a variety of exercise positions, such as those promoting strength, endurance, and flexibility

**Week 5. Taking medication**

- Insulin group:
Myths and facts about insulin injections
Introduction to different types of insulin and their durations of use
- Oral drugs group:
Directions and reminders for taking medication
Tips on what to do if they forget to take their medication

**Week 6. Taking medication**

- Insulin group:
Tips on injection skills and equipment
Travel guidelines for people with diabetes
- Oral drugs group:
Myths and facts about taking oral drugs
Travel guidelines for people with diabetes

**Week 7. Monitoring**

- The importance of glycemic monitoring
- Appropriate times and frequency of monitoring
- The meaning of fasting glucose

**Week 8. Monitoring**

- Hypoglycemia and hyperglycemia symptoms
- Common causes of hypoglycemia and hyperglycemia
- Tips for preventing hypoglycemia and hyperglycemia

**Week 9. Reducing risk**

- Causes of diabetic foot
- Symptoms of diabetic foot
- Self-care skills for diabetic foot
- Tips for selecting appropriate shoes for people with diabetes

**Week 10. Reducing risk**

- Self-care skills when sick
- Diabetic retinopathy and eye exam reminder

**Week 11.** **Problem solving**

- Stress and diabetes
- Tips for biopsychosocial adjustment, including developing good hobbies and positive thinking

**Week 12. Problem solving**

- How to search for correct information about diabetes

## Results

The baseline characteristics of the 3 groups in our study are shown in Table 1. No significant differences were observed in terms of gender, marital status, antidiabetic therapy, eye problems, foot problems, hypertension, lung disease, heart disease, cancer, arthritis, smoking, or exercise behavior across the 3 groups; also, no significant differences were observed in the means of the duration of diabetes, HbA_1c_ level, BMI, or CES-D score. However, participants in the mobile-based intervention group (n=49) were significantly younger and had higher educational levels than those in the telephone-based group (n=91) and usual care group (n=91). Also, participants in the mobile-based intervention group had higher PAID scores than those in the other 2 groups at baseline. These factors were used as stratification variables in our subgroup analysis.

Table 2 presents the pretest and posttest HbA_1c_, CES-D, and PAID scores and the differences between them across the 3 groups. Participants in the mobile-based group showed significant improvement in their HbA_1c_ levels after the intervention (posttest to pretest: –0.40%, *P*=.04) and PAID scores (posttest to pretest: –5.16, *P*=.01). We further used the Kruskal-Wallis test to examine the differences in the main outcomes across the groups. Our results showed that changes in the participants’ HbA_1c_ levels in the mobile-based intervention group (posttest to pretest: –0.40%) were significantly higher than those in the telephone-based group (posttest to pretest: 0.10%) and the usual care group (posttest to pretest: 0.03%). Also, the absolute improvement of the PAID score in the mobile-based intervention group (posttest to pretest: –5.16) was significantly higher than that of the telephone-based group (posttest to pretest: –3.49) and the usual care group (posttest to pretest: –2.44).

Due to the significant differences shown in Table 1 and the possible confounding associated with age, educational level, and depression symptoms at baseline, we further conducted a sensitivity analysis that stratified the participants by age, educational level, and baseline PAID score (Table 3). It was found that for participants aged <60 years, diabetes distress was significantly reduced in both the mobile-based intervention group (score –7.45, *P*=.01) and the telephone-based intervention group (score –4.87, *P*=.008). For participants with educational levels ≥7 grades, diabetes distress was also significantly reduced in both the mobile-based intervention group (score –8.80, *P*=.001) and the telephone-based intervention group (score –7.44, *P*=.008). In addition, participants in the mobile-based intervention group experienced a greater reduction in diabetes distress than those in the telephone-based intervention group, regardless of subgroup: age <60 years (the differences in the PAID score were mobile –7.45; telephone –4.87; usual care –4.14) or education ≥7th grade (the differences in the PAID score were mobile –8.80; telephone –7.44; usual care –5.00). Furthermore, we found that if the participants’ PAID was above the average (3.75), diabetes distress was significantly reduced in the mobile-based (score –6.89, *P*=.005), telephone-based (score –9.01, *P*<.001), and usual care groups (score –7.90, *P*=.001) after the intervention. In addition, for participants with diabetes distress scores lower than the median, only the mobile-based intervention group exhibited significantly reduced HbA_1c_ levels (score –0.31%, *P*=.04).

**Table 1:** The baseline sociodemographic, clinical, and psycho-behavioral characteristics of the mobile-based, telephone-based, and usual care groups (N=231).

Characteristic			Mobile-based group (n=49)		Telephone-based group (n=91)		Usual care group (n=91)		*P* value	
**Sociodemographic variables**										
	Age (years), mean (SD)			58.6 6.0)		64.70 (8.30)		64.70 (9.50)		<.001
	Male gender, n (%)			32 (65)		50 (55)		44 (48)		.16
	**Education level,** **n (%)**									
		Grade 6 and below	1 (2)		37 (41)		35 (39)		<.001	
		Grades 7-12	22 (45)		32 (35)		35 (39)			
		Grade 13 and above	26 (53)		22 (24)		21 (23)			
	Married or partnered, n (%)			43 (88)		71 (78)		75 (82)		.16
**Diabetes and other clinical variables**										
	Duration of diabetes (years), mean (SD)			7.5 (6.1)		10.60 (8.60)		10.40 (8.18)		.10
	**Diabetes treatment,** **n (%)**									
		Diet and exercise	7 (14)		5 (6)		10 (11)		.11	
		Oral medication	31 (63)		58 (63)		71 (78)			
		Insulin	11 (23)		20 (23)		10 (11)			
	HbA_1c_^a^ level (%), mean (SD)			7.6 (1.5)		7.6 (1.6)		7.6 (1.3)		.94
	BMI (kilograms per square meter), mean (SD)			25.9 (3.7)		25.80 (3.30)		25.90 (4.40)		.10
	Eye problems, n (%)			4 (8)		7 (8)		10 (11)		.71
	Foot problems, n (%)			2 (4)		10 (11)		5 (6)		.22
	Hypertension, n (%)			19 (39)		49 (54)		45 (50)		.23
	Lung disease, n (%)			0 (0)		2 (2)		2 (2)		.15
	Heart disease, n (%)			9 (18)		11 (12)		7 (8)		.17
	Cancer, n (%)			2 (4)		6 (7)		8 (9)		.57
	Arthritis, n (%)			2 (4)		10 (11)		8 (9)		.38
**Psychobehavioral variables**										
	Smoking, n (%)			4 (8)		13 (14)		4 (4)		.07
	**Exercise,** **n (%)**									
		Never	4 (9)		18 (20)		15 (17)		.09	
		1-2 times or less than 90 minutes per week	16 (32)		17 (19)		13 (14)			
		More than 3 times and >90 minutes per week	29 (60)		56 (61)		63 (69)			
	CES-D^b^ score (0-30), mean (SD)			6.8 (5.1)		5.20 (3.90)		5.60 (4.80)		.14
	Diabetes-specific emotional distress score (PAID^c^, 0-100), mean (SD)			13.5 (12.4)		5.40 (7.40)		5.20 (9.30)		<.001

^a^HbA_1c_: hemoglobin A1_c_

^b^CES-D: Center for Epidemiological Studies Depression Scale

^c^PAID: Problem Areas in Diabetes Scale

**Table 2:** Pretest and posttest HbA_1c_, CES-D, and PAID scores across the 3 groups and the differences between them.

Variable		Mobile-based group		Telephone-based group		Usual care group		Kruskal-Wallis test	
		Mean (SD)	*P* value^a^	Mean (SD)	*P* value^a^	Mean (SD)	*P* value^a^	Chi-square (*2*)	*P* value
**HbA_1c_^b^(%)**									
	Pretest	7.8 (1.49)	.04	7.64 (1.56)	.38	7.67 (1.28)	.78	8.3	.02
	Posttest	7.18 (1.05)		7.74 (1.47)		7.70 (1.18)			
	Difference^c^	–0.40 (1.24)		0.10 (1.12)		0.03 (1.1)			
**CES-D^d^ score**									
	Pretest	6.78 (5.08)	.31	5.19 (3.91)	<.01	5.59 (4.75)	.03	4.0	.13
	Posttest	7.95 (5.76)		3.84 (2.40)		4.47 (3.09)			
	Difference^c^	0.98 (6.14)		–1.34 (4.16)		–1.12 (4.86)			
**PAID^e^ score**									
	Pretest	13.52 (12.40)	.01	5.44 (7.39)	<.01	5.24 (5.29)	.03	7.6	.02
	Posttest	9.31 (9.79)		2.06 (6.14)		2.90 (4.79)			
	Difference^c^	–5.16 (12.58)		–3.49 (9.97)		–2.44 (10.60)			

^a^Calculated using the Kruskal-Wallis test.

^b^HbA_1c_: hemoglobin A1_c_.

^c^Difference: posttest value – pretest value.

^d^CES-D: Center for Epidemiological Studies Depression Scale.

^e^PAID: Problem Areas in Diabetes Scale.

**Table 3:** Sensitivity analysis comparing the intervention effects in the different subgroups.

Subgroup and effect		Mobile-based group			Telephone-based group			Usual care group			Kruskal-Wallis test	
		n	Score	*P* value	n	Score	*P* value	n	Score	*P* value	Chi-square (*2*)	*P* value
**Age ≥60 years (n=138)**												
	ΔHbA_1c_^a^ (%)	19	–0.42	.09	61	0.25	.03	58	0.08	.62	6.52	.04
	ΔCES-D^b^		2.35	.21		–1.44	.01		–1.11	.09	6.43	.04
	ΔPAID^c^		–2.06	.49		–2.82	.04		–1.49	.15	3.93	.14
**Age <60 years (n=93)**												
	ΔHbA_1c_ (%)	30	–0.38	.19	30	–0.19	.51	33	–0.05	.76	1.27	.53
	ΔCES-D		–0.04	.97		–1.14	.12		–1.16	.19	0.36	.84
	ΔPAID		–7.45	.01		–4.87	.008		–4.14	.11	4.62	.10
**Education≥7 grades (n=69)**												
	ΔHbA_1c_ (%)	26	–0.25	.31	22	0.12	.46	21	0.08	.55	1.71	.42
	ΔCES-D		0.09	.94		–0.71	.26		0.00	1.000	0.14	.93
	ΔPAID		–8.80	.001		–7.44	.008		–5.00	.06	3.24	.20
**Education<7 grades (n=162)**												
	ΔHbA_1c_ (%)	23	–0.57	.07	69	0.10	.50	70	0.02	.91	8.32	.02
	ΔCES-D		2.18	.20		–1.54	.007		–1.45	.02	4.24	.12
	ΔPAID		–0.22	.95		–2.26	.05		–1.70	.18	4.61	.10
**PAID mean score ≥3.75 (n=34)**												
	ΔHbA_1c_ (%)	17	–0.41	.08	6	0.15	.33	11	–0.05	.78	3.75	.15
	ΔCES-D		1.27	.28		–1.23	.09		–2.16	.01	4.36	.11
	ΔPAID		–6.89	.005		–9.01	<.0001		–7.90	<.001	1.68	.43
**PAID mean score <3.75 (n=197)**												
	ΔHbA_1c_ (%)	32	–0.31	.04	85	0.06	.73	80	0.09	.59	4.78	.09
	ΔCES-D		–0.43	.74		–1.44	.01		–0.35	.58	2.13	.35
	ΔPAID		3.04	.10		1.78	.16		1.62	.009	4.80	.09

^a^HbA_1c_: hemoglobin A_1c_.

^b^CES-D: Center for Epidemiological Studies Depression Scale.

^c^PAID: Problem Areas in Diabetes Scale.

## Discussion

### Principal Findings

This study examined the effectiveness of a mobile-based intervention on the glycemic control of people with diabetes, depressive symptoms, and diabetes-specific distress in comparison with telephone-based intervention and usual care. The results provide evidence that the mobile-based intervention has the greatest potential to improve glycemic control and reduce diabetes-specific distress among the 3 interventional methods. For participants in the mobile-based group who were less than 60 years of age or who had higher educational levels at baseline, their distress scores decreased more dramatically than those of the participants in the other 2 groups after the intervention. In addition, for participants with distress scores below the average at baseline, those who received the intervention based on the social and communication app LINE had significantly improved HbA_1c_ values, which was not observed in the telephone-based group or usual care group.

Previous studies evaluating technology-enabled self-management education mostly used web-based structured diabetes education, time-consuming nurse coaching, and online educational discussion groups to aid diabetes self-management [8]. Although these approaches can reduce depressive symptoms and diabetes-related distress, they are labor-intensive and may be expensive. In the present study, we utilized a patient-centered approach and a freely available social app. We sent text and photographic messages based on the AADE’s 7 indicators for diabetes self-management to middle-aged and older adults through the free social and communication app LINE, which the participants were familiar with. In addition, we provided social support, problem solving, and communication with professionals through the LINE app. With figure-rich messages for diabetic education, the LINE app is suggested to be an effective platform to execute diabetes education and support; it may improve diabetes glycemic control and reduce diabetes-related distress more than usual care, supporting previous studies indicating the strength of mobile phones in this area [38]. The strengths of social and communication apps include their ability to send multimedia messages and free video calls; also, using mobile-based devices, participants can browse their messages repeatedly and magnify the image-based messages, which addresses the decline in cognitive functioning required for older adults to manage their disease. Also, the bright colors and clear font size in the photo messages are age-friendly and help older adults avoid eye fatigue [39]. We found that the participants not only experienced decreased diabetes distress but also had increased glycemic control performance.

The success of the application of the LINE app in diabetes care in middle-aged and older adults in Taiwan may be due to their cultural background of the participants and the readily accessible and affordable health care system. As addressed in previous literature, preferences for mobile apps developed for diabetes management are different in different countries; Americans prefer apps that support their decision-making, while Chinese and Middle Eastern residents prefer app-based communication functions [40,41]. Korean people also benefited from an automated self-measured blood glucose upload built-in diabetes self-management app with physician response once a week [42]. In brief, patients in Asia and the Middle East prefer to seek advice from their health care teams directly through the communication app, while patients in western countries prefer to solve problems by themselves if the diabetes-specific app can provide adequate resources to support their decision-making. On the other hand, the language of most diabetes apps is English (85.4%); this limits the selection of diabetes apps in Taiwan. Additionally, patients prefer free apps to paid apps [43]. In this era of knowledge explosion, patients can search for diabetes management articles online; however, they may not recognize whether the information in these articles is correct [44]. There is no one app that is suitable for all patients with diabetes. Development of diabetic self-management mobile apps in Taiwan also requires much user feedback [45]. However, even if a dedicated developed app that contains all suggested functions and articles beneficial for diabetes self-management were accessible, patients would not use all the built-in functions to enhance their self-care behavioral skills [46]. For the reasons addressed above, the application of LINE for diabetes care in Taiwan is appropriate in many aspects; it can be used to communicate with health care teams, can provide carefully selected articles that assist diabetes self-management, and is freely available. We also used a strategy of not providing too many messages at a time; therefore, participants could absorb the diabetes self-management knowledge in steps during the 12-week intervention period.

In the present study, we found when the diabetes-related distress scores for the participants were higher before the intervention, their diabetes distress was significantly reduced in both the mobile-based and telephone-based groups. Increasing numbers of studies are focusing on mobile-related devices or applications rather than telephone-based interventions due to the fact that such devices and applications are cost-effective, can reach large populations, suggest promising outcomes, and have expanded rapidly in the past decade [47-50]. This study also suggests that mobile-related applications are more cost-effective than telephone-based interventions and are very efficient. Previous studies have provided services that deliver interventions, including brief psychological therapies, mental health assessments, psychotropic medications, and social support, enhanced by patient-led case conferences aiming to optimize diabetes care. The results of these studies indicate a significant reduction in HbA_1c_ of 3.5%, which was associated with reductions at 1-year follow-up [51]. Our study found that for the mobile-based intervention group, when we focused on people with diabetes whose diabetes distress scores were lower than the median score, there was a significant reduction in HbA_1c_ scores during the 12-week period. This can be explained by the fact that information seeking is one of the most important elements of coping strategies of chronic illness among patients who have comorbid depression [52]; immediate responses and conversation privacy are additional strengths of mobile-based interventions.

In this study, we found that the decrease of the PAID score with the mobile-based intervention was greater when the participants were younger and when their education levels were higher. This finding echoes those of previous studies indicating that people newly diagnosed with diabetes are willing to participate in self-management programs where their medical outcomes are being effectively targeted [53,54].

### Limitations

There were some limitations of this study. First, this study had a small sample size. Second, it did not randomly allocate participants to the 3 interventions. However, we compared the mobile-based group with the telephone-based and usual care groups and found manageable differences. Third, this study did not evaluate changes in behavior-related variables. Future studies are suggested to test the effects of this intervention in a broader sample and to evaluate both its behavioral and long-term effects.

### Conclusion

Existing free social and communication apps are effective to improve glycemic control and reduce diabetes-specific distress in older adults with diabetes. For participants younger than 60 years, with higher educational levels, or with higher diabetes-related distress at baseline, the social and communication app reduced their distress scores more dramatically than those of participants in the other 2 groups after the intervention. In addition, for participants whose distress scores were below average at baseline, the social and communication app significantly improved their glycemic control.

## Appendix

Multimedia Appendix 1 Sample messages.

## Abbreviations

AADE: American Association of Diabetes Educators
HbA_1c_: hemoglobin A_1c_
PAID: Problem Areas in Diabetes Scale
CES-D: Center for Epidemiological Studies Depression Scale
