# Supplementary material for: Comparing a Social and Communication App, Telephone Intervention, and Usual Care for Diabetes Self-Management: 3-Arm Quasiexperimental Evaluation Study
Source: JMIR Mhealth Uhealth. 2020 Jun 2;8(6):e14024. doi: 10.2196/14024 (PMC7298636; doi:10.2196/14024)
Supplement: Multimedia Appendix 1 [file mhealth_v8i6e14024_app1.docx]

Multimedia Appendix. Sample messages

| Weekly topic | Sample messages | |
| --- | --- | --- |
| Week 1. Healthy eating | 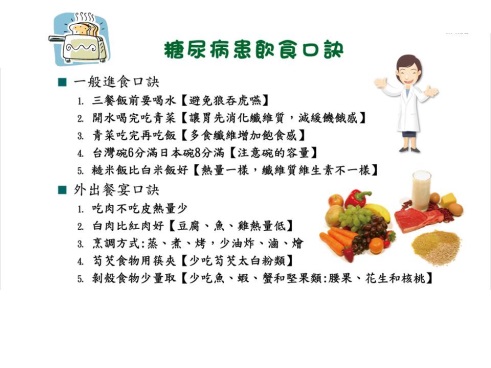 | 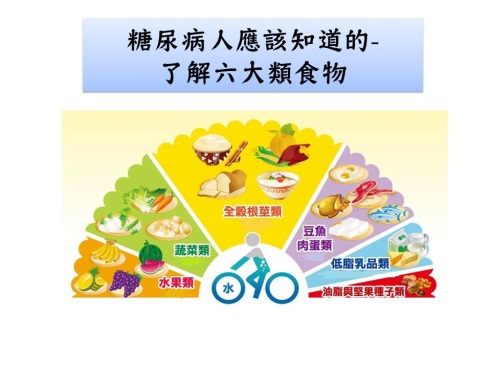 |
| Week 2. Healthy eating | 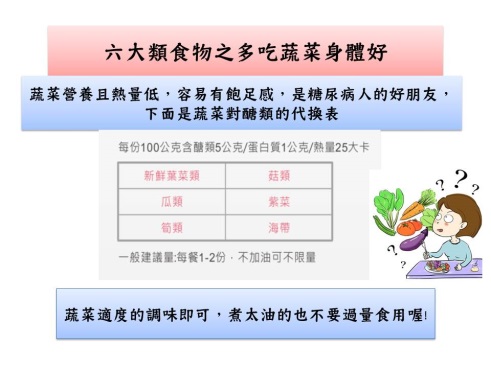 | 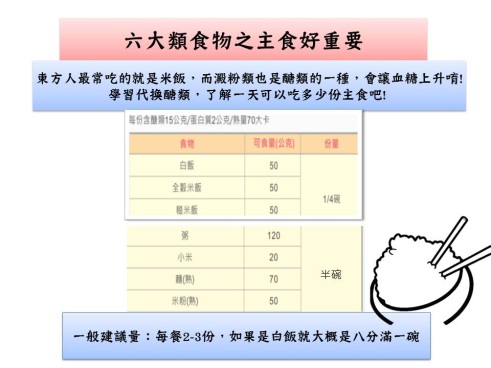 |
| Week 3. Being active | 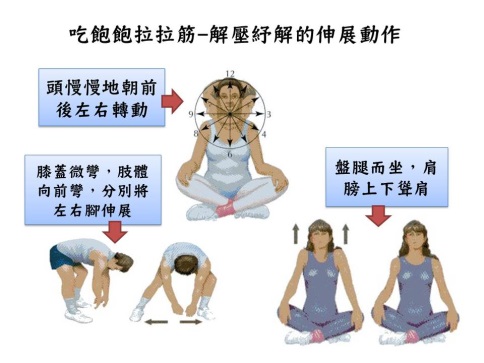 | 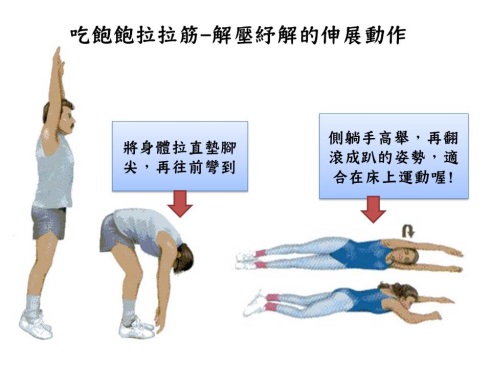 |
| Week 4. Being active | 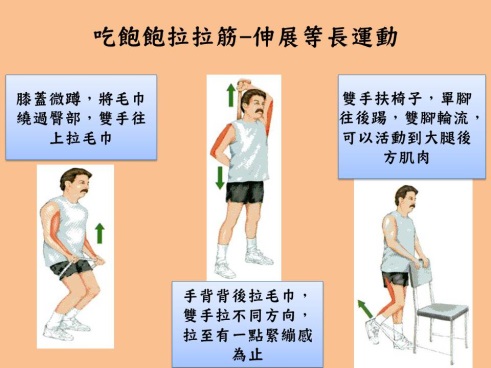 | 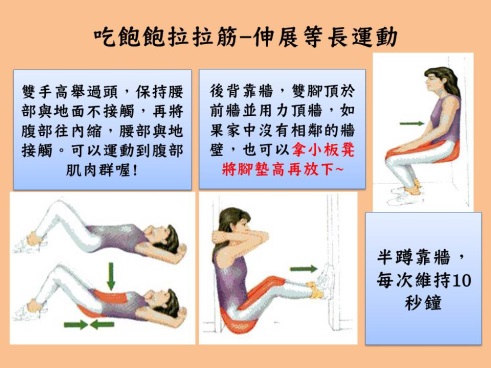 |
| Week 5. Taking Medication | 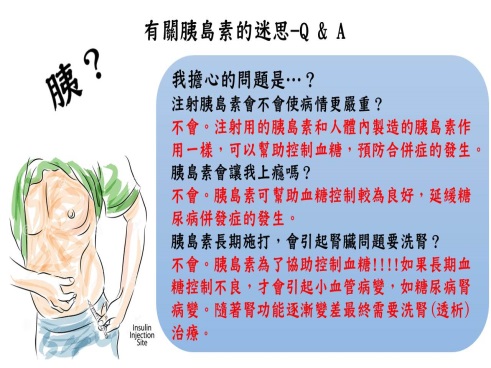 | 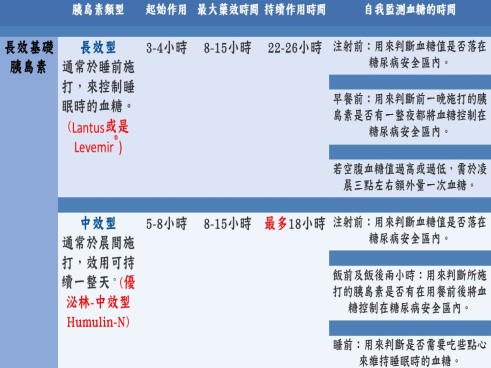 |
| Week 6. Taking Medication | 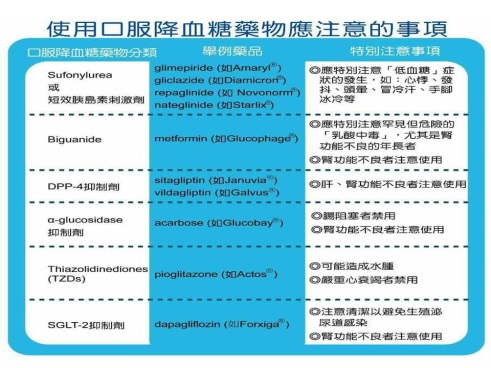 | 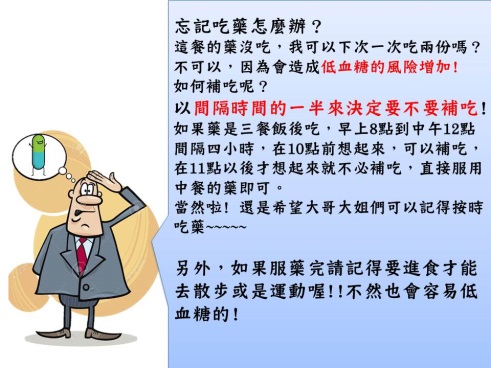 |
| Week 7. Monitoring | 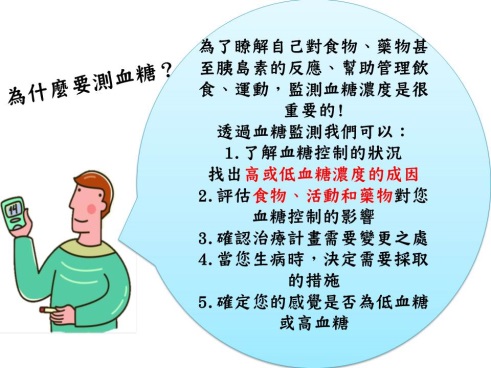 | 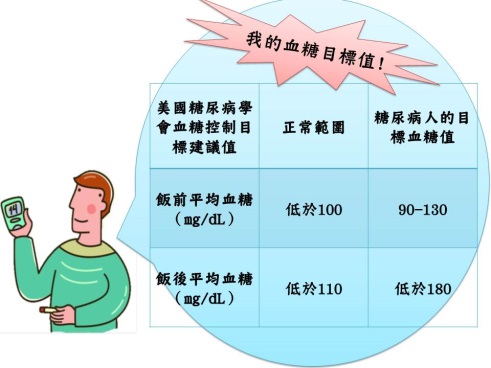 |
| Week 8. Monitoring | 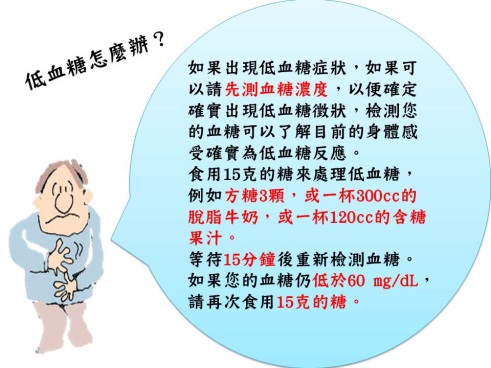 | 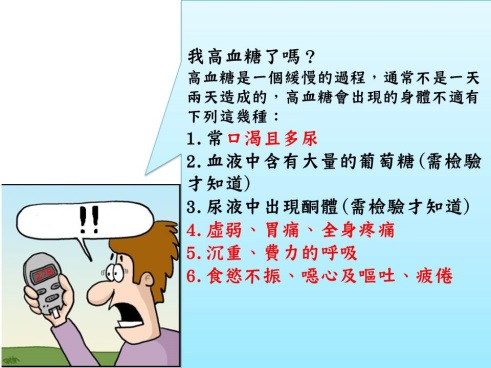 |
| Week 9. Reducing risk | 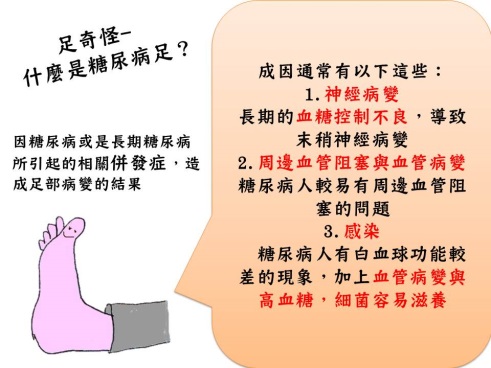 | 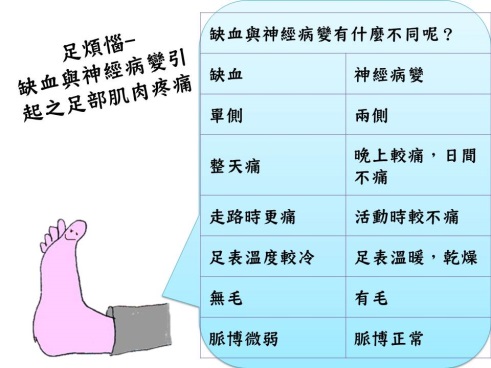 |
| Week 10. Reducing risk | 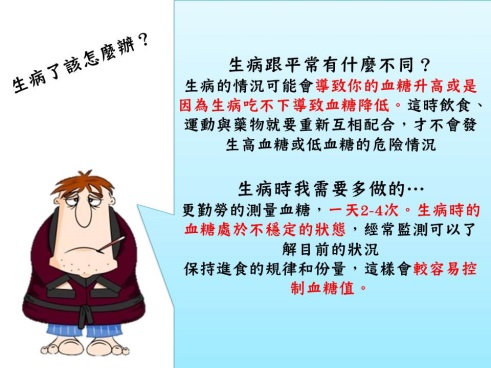 | 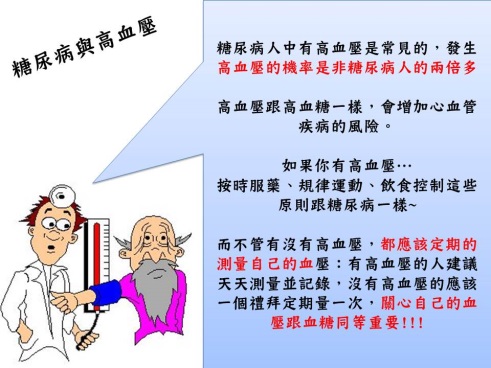 |
| Week 11. Problem Solving | 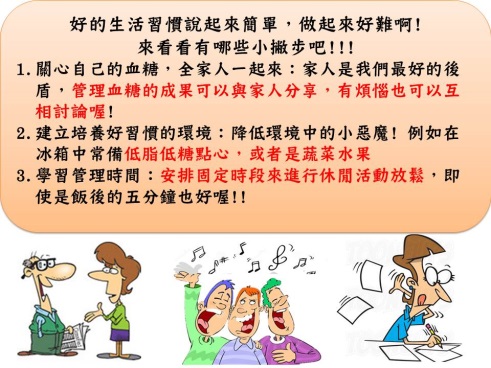 | 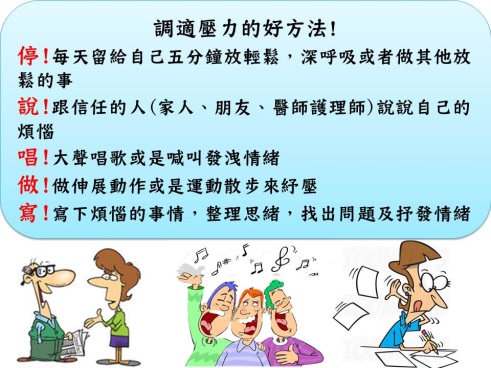 |
| Week 12. Problem Solving | 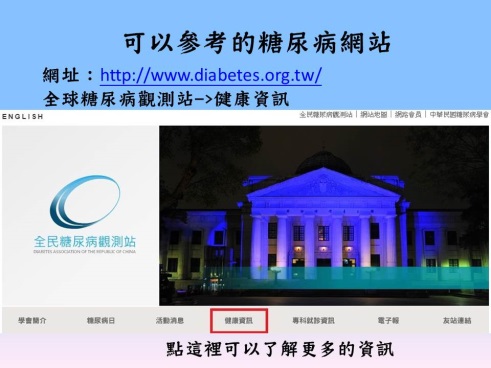 | 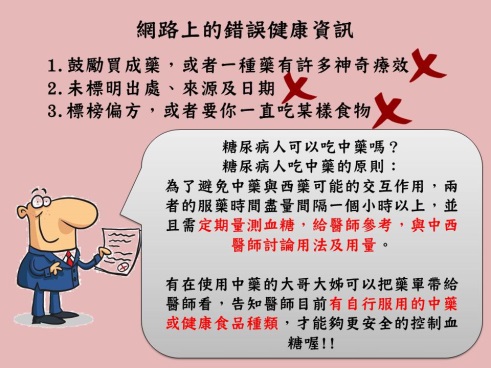 |
|  | | |
